# Protective Effect of Pyrogallol-Phloroglucinol-6,6-Bieckol from *Ecklonia cava* on Monocyte-Associated Vascular Dysfunction

**DOI:** 10.3390/md16110441

**Published:** 2018-11-09

**Authors:** Seyeon Oh, Myeongjoo Son, Hye Sun Lee, Hyun-Soo Kim, You-Jin Jeon, Kyunghee Byun

**Affiliations:** 1 Functional Cellular Networks Laboratory, Lee Gil Ya Cancer and Diabetes Institute, Gachon University, Incheon 21999, Korea; seyeon8965@gachon.ac.kr (S.O.); mjson@gachon.ac.kr (M.S.); 201740538@gc.gachon.ac.kr (H.S.L.); 2 Department of Anatomy & Cell Biology, Graduate School of Medicine, Gachon University, Incheon 21936, Korea; 3 Department of Health Sciences and Technology, GAIHST, Gachon University, Incheon 21999, Korea; 4 Department of Marine Life Science, Jeju National University, Jeju 63243, Korea; gustn783@naver.com

**Keywords:** poor blood circulation, *Ecklonia cava*, phlorotannins, pyrogallol-phloroglucinol-6,6-bieckol, functional ingredients, endothelial cell death, vascular smooth muscle cell proliferation and migration, inflammation

## Abstract

*Ecklonia cava* (*E. cava*) can alleviate vascular dysfunction in diseases associated with poor circulation. *E. cava* contains various polyphenols with different functions, but few studies have compared the effects of these polyphenols. Here, we comparatively investigated four major compounds present in an ethanoic extract of *E. cava*. These four major compounds were isolated and their effects were examined on monocyte-associated vascular inflammation and dysfunctions. Pyrogallol-phloroglucinol-6,6-bieckol (PPB) significantly inhibited monocyte migration in vitro by reducing levels of inflammatory macrophage differentiation and of its related molecular factors. In addition, PPB protected against monocyte-associated endothelial cell death by increasing the phosphorylations of PI3K-AKT and AMPK, decreasing caspase levels, and reducing monocyte-associated vascular smooth muscle cell proliferation and migration by decreasing the phosphorylations of ERK and AKT. The results of this study show that four compounds were effective for reduction of monocyte-associated vascular inflammation and dysfunctions, but PPB might be more useful for the treatment of vascular dysfunction in diseases associated with poor circulation.

## 1. Introduction

Weight gain has been intimately associated with diseases associated with poor circulation, such as stroke, atherosclerosis and high blood pressure [1,2]. Most obese individuals have elevated blood levels of glucose, low-density lipoprotein (LDL) or free fatty acids (FFA), and these changes can alter blood functions and blood vessel construction.

In blood vessel walls, endothelial cells (ECs) and vascular smooth muscle cells (VSMCs) influence vessel tone. ECs form vessel barriers, regulating blood flow and inflammatory response, whereas VSMCs have proliferative, contractile and biosynthetic roles in vessel walls. Alterations in the differentiated states of these cells play critical roles in the pathogeneses disease associated with poor circulation. Saturated free fatty acids (FFA), elevated glucose or LDL lead to EC and VSMC dysfunction. Furthermore, these abnormal changes induce bioavailable nitric oxide (NO) deficiency, reduce vascular relaxation, induce the overproductions of growth factors, increase adhesion and inflammatory molecule expressions, induce the generation of reactive oxygen species (ROS) in ECs [3,4,5], adversely influence glucose metabolism, and promote the abnormal proliferation and migration of VSMCs [6,7].

High glucose, LDL and FFA can also indirectly affect ECs and VSMCs via the inflammation induction of monocytes. Obesity affects the activations of circulating monocytes. In particular, high glucose levels increase monocyte adhesion and trans-endothelial migration by activating the AKT-GSK axis [8], which leads to the inductions of inflammatory factors, such as tumor necrosis factor-alpha (TNF-α), monocyte chemoattractant protein-1 (MCP-1), interleukin-beta (IL-1β) and Toll-like receptors (TLRs) via oxidant stress [9,10,11].

Edible marine plants have emerged as a potential source of bioactive compounds for the developments of cosmeceutical ingredients [12]. *Ecklonia cava* (*E. cava*) is an edible marine brown alga, and it is one of nature’s richest sources of phlorotannins, and phlorotannin derivatives which do not exist in land-originating plants. The phlorotannins are a sub-classification of polyphenolic compounds that are confirmed by dibenzo-1,4-dioxin backbone which is this backbone linkage can make the structure tight and strongly interact with various biological molecules [13,14]. *E. cava* extracts have been shown to suppress the production of inflammatory cytokines and the activation of NF-κB in lipopolysaccharide (LPS) challenged human ECs and to reduce vascular inflammation by preventing oxidation [13,15].

Some studies have shown these compounds have different beneficial effects of various *E. cava* phlorotannins, but the efficacies of these compounds have not been previously compared. In a previous study [16], we successfully isolated four phlorotannins from an ethanoic extract of *E. cava*, that is, dieckol, 2,7-phloroglucinol-6,6-bieckol (PHB), phlorofucofuroeckol-A (PFFA), and pyrogallol-phloroglucinol-6,6-bieckol (PPB), by centrifugal partition chromatography. In the present study, we sought to determine which compound most effectively inhibits monocyte migration and differentiation to inflammatory macrophages and monocyte-associated vascular cell dysfunction in vitro.

## 2. Results and Discussion

### 2.1. Structures of the Four Compounds Isolated from E. cava

**Figure 1:**
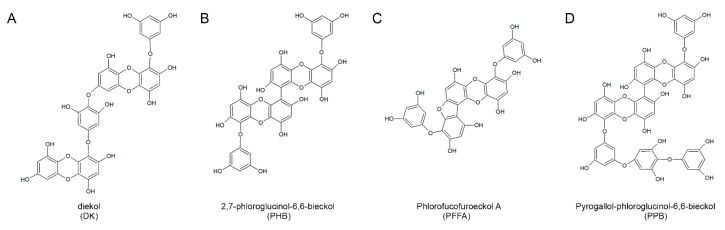
The chemical structures of the four major compounds isolated from *E. cava*. (**A**–**D**) Chemical structures of dieckol (DK), 2,7-phloroglucinol-6,6-bieckol (PHB), phlorofucofuroeckol-A (PFFA), and pyrogallol-phloroglucinol-6,6-bieckol (PPB).

The four compounds were isolated and purified using centrifugal partition chromatography in one step [16]. The peaks a-d on the high-performance liquid chromatography (HPLC) shown in Figure S1 were assigned to DK, PHB, PFFA and PPB, respectively by mass spectrometry analysis (Figure S2). They show a single peak in the HPLC chromatogram and had a purity of 90% or more. Our previous study shown the 4 compounds identified using ^1^H NMR and ^13^C NMR and HPLC–DAD–ESI/MS (negative ion mode) analyses [16] and each chemical structure shown in Figure 1. Previous studies on various biological properties of phlorotannins including anti-oxidant [17], anti-inflammation [18], anti-neurodegeneration [19], anti-cancer [20,21], and anti-cardiovascular diseases [22] of *E. cava* extract have shown. Among numerous properties, anti-oxidant activities of *E. cava* phlorotannins extract on reactive oxygen species (ROS) have shown it exhibits radical scavenging activity against oxidized low-density lipoprotein (ox-LDL), 1,1-diphenyl-2-picrylhydrazyl (DPPH) radicals, and peroxynitrite [14,16,17] and these anti-oxidant activities closely related with other beneficial effects of *E. cava*.

Interestingly, the difference in anti-oxidant effect between various *E. cava* phlorotannins is related to the number of hydroxyl groups present. According to a study by Li and colleagues, dieckol and 6,6′-bieckol (more than 10 OH groups) had higher anti-oxidant efficacy than phloroglucinol and eckol (less than 10 OH groups) [14]. The PPB used in this study is also expected to have anti-inflammatory effects, including monocyte migration and macrophage polarization, because of the presence of 15 OH groups.

### 2.2. Analysis of the Effects of 4 Compounds on Monocyte Migration and Macrophage Polarization

**Figure 2:**
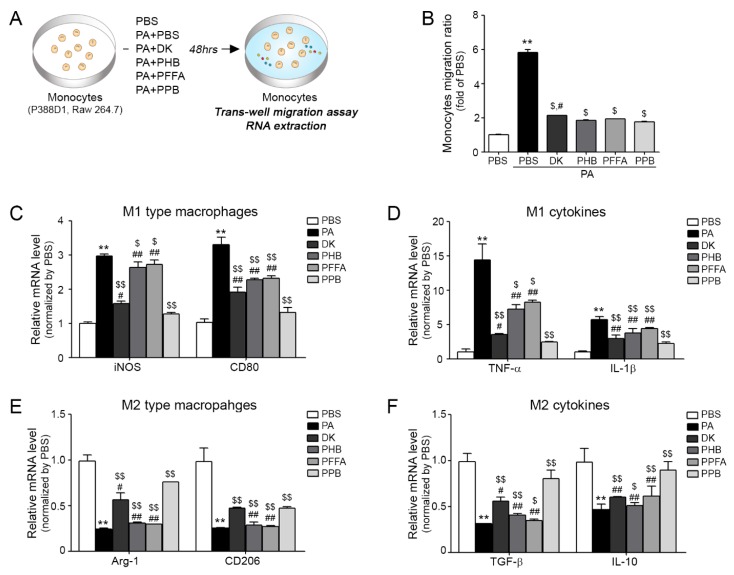
Inhibitory effects of PPB on monocyte polarization and related cytokines and EC dysfunction. (**A**) Illustration showing the palmitic acid conjugated bovine serum albumin (PA-BSA)-treated monocyte trans-migration model. (**B**) Migrating monocytes levels in 4 compounds with PA-BSA as determined by the trans-well migration assay. (**C**,**D**) mRNA expression levels of M1 type macrophages (inducible nitric oxide synthase (*iNOS*) and *Cd80*) and M2 type macrophages (arginase-1 (*Arg-1*) and *Cd206*) as determined by quantitative real-time polymerase chain reaction (qRT-PCR). (**E**,**F**) mRNA expression levels of M1 related cytokines (tumor necrosis factor-alpha (*TNF-α*) and interleukin-beta (*IL-1β*)) and M2 related cytokines (transforming growth factor beta 1 (*TGF-β*) and interleukin-10 (*IL-10*)) by qRT-PCR. Kruskal–Wallis tests were used to determine differences between groups and post-hoc comparisons were made with the Mann–Whitney U test **, *p* < 0.01, ***, *p* < 0.001, vs. PBS; $, *p* < 0.05, $$, *p* < 0.01, vs. PA-BSA; #, *p* < 0.05, ##, *p* < 0.01, vs. PA-BSA with PPB, DK; dieckol, PHB; 2,7-phloroglucinol-6,6-bieckol, PFFA; phlorofucofuroeckol-A, PPB; pyrogallol-phloroglucinol-6,6-bieckol.

In diseases associated with inadequacy of blood flow in organs [23], monocyte migration is important and closely related to vascular inflammation. Figure 2A provides a schematic of the inhibitory effects of these four compounds on palmitic acid conjugated bovine serum albumin (PA-BSA) induced monocyte trans-migration and macrophage polarization (Figure 2A). Experiments were performed on two monocyte cell lines (P388D1 and Raw 264.7). Numbers of trans-migrating monocytes were greatest for PA-BSA-treated monocytes, and all four compounds significantly reduced numbers of migrating cells (Figure 2B) and the results were similar in Raw 264.7 (Figure S3A). Trans-migrating monocytes differentiated to macrophages of the pro-inflammatory (M1 type macrophages) or anti-inflammatory (M2 type macrophages) types (Figure 2C–F). Furthermore, PA-BSA-treated monocytes contained elevated levels of inflammatory factors, including inducible nitric oxide synthase (iNOS), CD80, TNF-α and interleukin-1β (IL-1β) (Figure 2C,D) and low levels of anti-inflammatory like arginase-1 (Arg-1), CD206, transforming growth factor beta 1 (TGF-β) and interleukin-10 (IL-10) (Figure 2E,F) and the results were similar in Raw 264.7 (Figure S3B–E). Interestingly, when monocytes were treated with four compounds with PA-BSA and these inductions were reduced, PPB had the greatest effect. As well as its anti-oxidant effects, *E. cava* extract has anti-inflammatory effects. For example, an ethanoic extract of *E. cava* was found to contain large amounts of phlorotannins and to inhibit the productions of prostaglandin-E2 (PGE2) and nitric oxide (NO) and suppress cyclooxygenase-2 (COX-2) and iNOS expressions in LPS-stimulated Raw 264.7 cells [24]. In inflammatory lung diseases, *E. cava* extract was found to significantly reduce inflammatory reactions, such as eosinophil migration to lungs, inflammatory cell and cytokine increases, and to reduce airway epithelial hyperplasia, lung fibrosis and smooth muscle cell thickness [14,15,17,25].

### 2.3. Effects of Four Compounds on Monocyte-Induced Endothelial Cell Death

**Figure 3:**
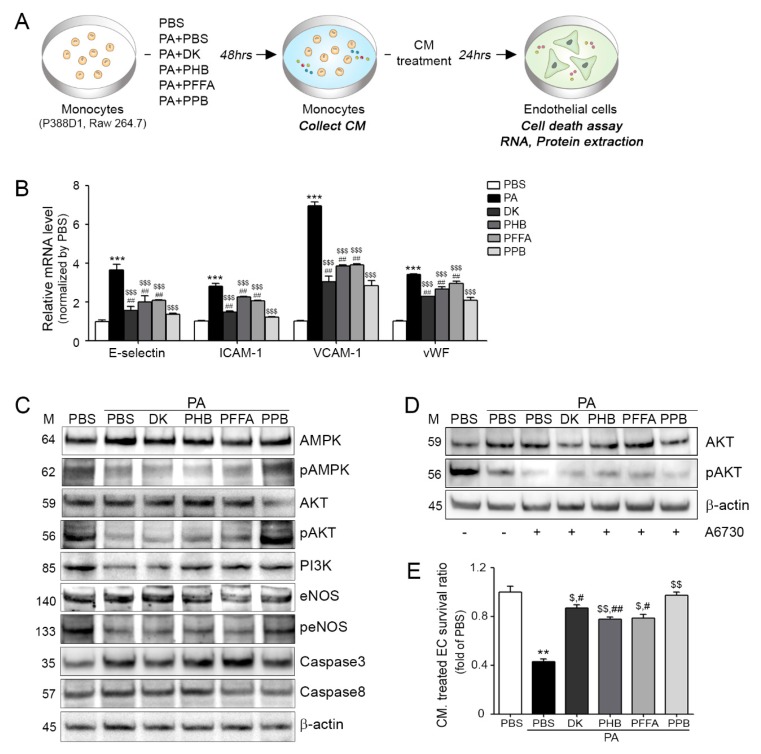
Prevention of monocyte-induced endothelial cell death by DK, PHB, PFFA or PPB. (**A**) Endothelial cells (ECs) were treated with conditioned medium (CM) collected from PA-BSA induced transmigrating monocytes. (**B**) mRNA expression levels of adhesion molecules (*E-selectin*, *ICAM-1*, *VCAM-1* and *vWF*) in CM treated ECs were measured by qRT-PCR. (**C**) Protein levels of cell-death related molecules, that is, AMPK, pAMPK, AKT, pAKT, PI3K eNOS, peNOS, Caspase 3, and Caspase 8 in CM treated endothelial cells were determined by western blotting. (**D**) The AKT inhibitor (A6370) was treated to monocyte and collected CM was treated EC. The ECs determined by western blotting. (**E**) Survival levels of CM treated ECs were measured using a cell survival assay. Kruskal–Wallis tests were used to determine differences between groups and post-hoc comparisons were made with the Mann–Whitney U test. **, *p* < 0.01, ***, *p* < 0.001, vs. PBS; $, *p* < 0.05, $$, *p* < 0.01, $$$, *p* < 0.001, vs. PA-BSA; #, *p* < 0.05, ##, *p* < 0.01 vs. PA-BSA with PPB, DK; dieckol, PHB; 2,7-phloroglucinol-6,6-bieckol, PFFA; phlorofucofuroeckol-A, PPB; pyrogallol-phloroglucinol-6,6-bieckol.

**Figure 4:**
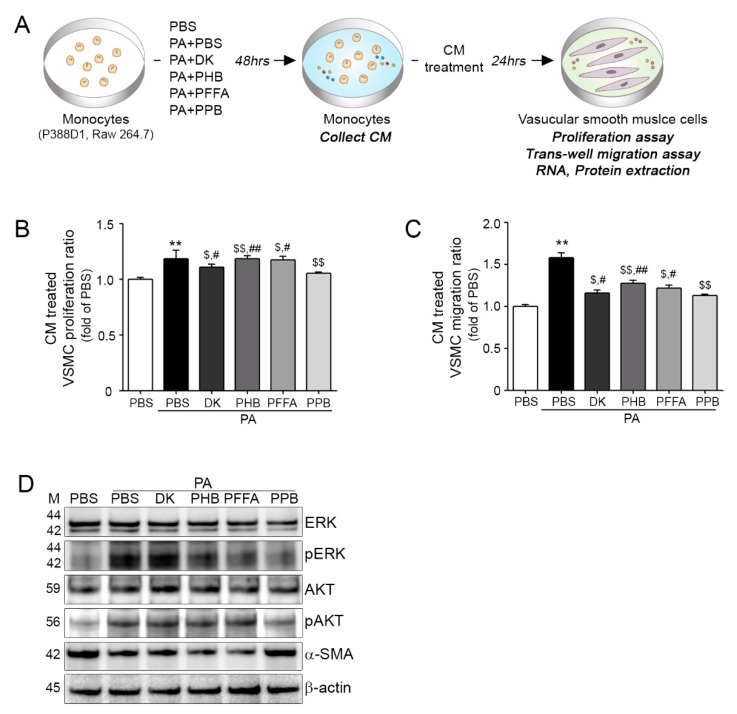
DK, PHB, PFFA or PPB inhibited monocytes migration and prevented monocyte-associated vascular smooth muscle cell proliferation and migration. (**A**) Illustration of the CM-induced vascular smooth muscle cells (VSMCs) proliferation and trans-migration model. (**B**) VSMC proliferation after CM treatments were measured using a proliferation assay. (**C**) Trans-migrating VSMC numbers were measured using a trans-migration assay. (**D**) Protein levels of proliferation and migration related molecules, that is, ERk, pERK, AKT, pAKT, α-SMA in CM treated VSMCs were determined by western blotting. Kruskal–Wallis tests were used to determine differences between groups and post-hoc comparisons were made with the Mann–Whitney U test. **, *p* < 0.01, vs. PBS; $, *p* < 0.05, $$, *p* < 0.01, vs. PA-BSA; #, *p* < 0.05, ##, *p* < 0.01, vs. PA-BSA with PPB, DK; Dieckol, PHB; 2,7-phloroglucinol-6,6-bieckol, PFFA; phlorofucofuroeckol-A, PPB; pyrogallol-phloroglucinol-6,6-bieckol.

The inhibiting effects of four compounds on PA-BSA treated ECs and VSMCs dysfunctions induced by monocytes are summarized in Figure 3 and Figure 4. Monocytes were treated four compounds with PA-BSA respectively, and then each conditioned medium (CM) from the four compounds treated monocytes was incubated with ECs or VSMCs for 24 h (Figure 3A and Figure 4B). Adhesion molecule expressions (E-selectin, intercellular adhesion molecule 1; ICAM-1, vascular cell adhesion molecule 1; VCAM-1 and von Willebrand factor; vWF) in ECs were significantly higher when they were treated with PA-BSA CM than BSA CM, but the expression was significantly lowest when ECs were treated with PPB CM (Figure 3B). The various adhesion molecules are related to vascular inflammation, and these molecules are regulated by mast cells, macrophages, and neutrophils, which also secrete pro-inflammatory cytokines, such as TNF-α, interferon gamma (IFN-γ), and IL-6. These pro-inflammatory cytokines induce the expressions of adhesion molecules in ECs and recruit leukocytes, which are important components of the pathogenesis of vascular inflammation [26,27]. Adhesion molecules are also related to EC survival [21]. In addition to the abovementioned adhesion molecule changes, all four compounds improved the survival ratios of PA-BSA CM treated ECs, and PPB CM treated cells had the lowest levels of caspases 3 and 8 and it was related with the phosphorylated PI3K-AKT-eNOS and AMPK signaling pathways (Figure 3C,E and Figure S4). In addition, although the AKT inhibitor A6730 was treated, the expression of pAKT was increased in the single compounds treated group (Figure 3D). In ECs, the PI3K-AKT pathway is essential for mediating cell survival, migration, proliferation, and angiogenesis [28,29]. In particular, high glucose-induced EC apoptosis depends on Akt de-phosphorylation and activation of the PI3K/AKT/eNOS signaling pathway protects ECs from apoptosis [30].

### 2.4. Effects of All Four Compounds on Monocyte-Induced VSMC Proliferation and Migration

DK, PHB, PFFA and PPB CM treated VSMCs proliferated and migrated significantly less than PA-BSA CM treated VSMCs, and PPB CM was related with phosphorylations of the AKT and ERK pathways (Figure 4B–D), and the results were similar in Raw 264.7 (Figure S5). In addition, PA-BSA CM treated VSMCs had the highest α-SMA levels, and PPB CM had greatest effect, suggesting VSMCs would be closer to the contractile phenotype (Figure 4D and Figure S5C). Phenotype switching of VSMCs is important for the maintenance of vascular tone and alpha-smooth muscle actin (α-SMA) promotes the synthetic phenotype. In previous studies, higher expression of α-SMA in PA-BSA than in BSA treated VSMCs was found to reduce the contractile phenotype and increase proliferation and migration rates via the AKT and ERK pathways [31,32,33]. VSMCs can perform both contractile and synthetic functions, which are associated with the maintenance of vascular tone. The synthetic VSMCs phenotype has characteristics that include increased proliferation and migration rates, extensive ECM degradation/synthesis abilities, and an increased cell size, which is closely related with neo-intima hyperplasia formation [31,32,33,34].

## 3. Materials and Methods

### 3.1. Materials

#### 3.1.1. *E. cava* Extraction

*E. cava* powder (2.5 g) was soaked in 50% ethanol (100 mL) and stirred at 130 rpm for 1 h at room temperature. The mixture was then centrifuged at ~3667× *g* for 10 min, and the supernatant was filtered through 3M paper and concentrated under vacuum. The crude extract was stored at −20 °C until required.

#### 3.1.2. Isolation of Compounds from *E. cava* Extract

Compounds were isolated, as previously described [16]. Briefly, centrifugal partition chromatography (CPC) was performed using a two-phase solvent system comprised of n-hexane/ethyl acetate/methanol/water (2:7:3:7, *v*/*v*/*v*/*v*). The CPC column was first filled with the organic stationary phase and the mobile phase was pumped into the column in descending mode at the same flow rate used for separation (2 mL/min).

#### 3.1.3. Experimental Cell Models

To prepare PA-BSA, 2.267 g of fatty acid-free BSA (Sigma-Aldrich; St. Louis, MO, USA) was thawed in pre-warmed 100 mL of 150 mM NaCl. The mixture was stirred at 37 °C (no higher than 40 °C) in a water bath until completely dissolved. The BSA solution was from a filtered new bottle and it was stirred at 37 °C. While the BSA was being stirred in the water bath, 30.6 mg of Sodium palmitate was thawed in 150 mM NaCl 50 mL in a water bath at 70 °C.

The PA-BSA was divided into 5 mL portions and transferred to the BSA solution, stirred at 37 °C for 1 h, and adjusted to a final volume of 100 mL with 150 mM NaCl and pH 7.4 with 1N NaOH. The solution was stored −20 °C until required and thawed in a 37 °C water bath for 10 min prior to use.

### 3.2. Cell Culture and Treatment

#### 3.2.1. Monocytes

Monocytes (P388D1 cells) were purchased from ATCC (Washington, DC, USA). RPMI 1640 (Gibco; Grand island, NY, USA), 10% fetal bovine serum (FBS), 25 mM hydroxyethyl-piperazineethane-sulfonic acid buffer (HEPES) buffer and 1% penicillin-streptomycin were used as growth medium. To investigate the inhibitory effects of DK, PHB, PFFA and PPB in 0.25 mM PA-BSA treated monocytes, we used the same concentration (2.5 μg/mL) for a treatment time of 48 h. To collect conditioned medium (CM), monocytes were treated with PA-BSA with or without DK, PHB, PFFA or PPB for 48 h.

#### 3.2.2. Vascular Endothelial Cells (ECs)

ECs (SVEC 4–10 cells) were also purchased from ATCC. Dulbecco’s Modified Eagle’s medium (DMEM; Gibco) and 1% penicillin-streptomycin (Gibco) were used as growth medium.

#### 3.2.3. Vascular Aortic Smooth Muscle Cells (VSMCs)

VSMCs (MOVAS cells) were also obtained from ATCC. DMEM, 10% FBS and antibiotics G-418 were used as growth medium.

### 3.3. Extraction and Isolation

#### 3.3.1. RNA Extraction and cDNA Synthesis

The cells were homogenized in ice using a disposable pestle in 1 mL of RNisol (TAKARA; Kusatsu, Japan), and homogenates were added to 0.2 mL of chloroform, mixed, and centrifuged at 12,000× *g* for 15 min at 4 °C. Aqueous phases were collected, placed in cleaned tubes, mixed with 0.5 mL of isopropanol, and centrifuged using the same conditions. Isolated RNA was then washed with 70% ethanol and dissolved in 50 µL of diethyl pyrocarbonate (DEPC) treated water. To perform quantitative real-time polymerase chain reaction (qRT-PCR), cDNA was synthesized from 1 μg of total RNA using a Prime Script 1st strand cDNA Synthesis Kit (TAKARA, Japan).

#### 3.3.2. Protein Isolation

Cell proteins were extracted using the EzRIPA lysis kit (ATTO; Tokyo, Japan). Initially, tissues were homogenized with lysis buffer containing proteinase and phosphatase inhibitors and briefly sonicated for 10 s in a cold bath sonicator. After centrifuging at 14,000× *g* for 20 min at 4 °C, supernatants were collected and protein concentrations were determined using a Bicinchoninic acid assay kit (BCA kit; Thermo Fisher Scientific, Inc.; Waltham, MA, USA).

### 3.4. Monocyte Trans-Well Migration Assay

Monocytes were seeded at a density of 10^6^ per well onto 8-µm Transwell inserts (Thermo Fisher Scientific). The lower chamber was filled with 500 μL low serum medium containing DK, PHB, PFFA or PPB and 0.25 mM PA-BSA and incubated for 48 h at 5% CO_2_ incubator. Migration activities were evaluated using water-soluble tetrazolium salts (WST; Daeil Lab Service Co.; Seoul, Korea) and optical densities were measured.

### 3.5. Monocyte-Associated EC Viability Assay

To analyze monocyte-associated EC viability, 5000 ECs were seeded in the wells of a 96-well culture plate (Thermo Fisher Scientific) and incubated for 24 h in a 5% CO_2_ humidified incubator at 37 °C. The WST was mixed with serum free DMEM (1:9, *v*/*v*, 200 µL/well) and the mixture was incubated for 4 h in ECs. Optical densities were measured using a plate reader at 450 nm (Spectra max plus, Molecular devices).

### 3.6. Monocyte-Associated VSMC Proliferation Assay

To analyze monocyte-associated VSMC proliferation, VSMCs were seeded in a 96-well culture plate (Thermo Fisher Scientific Inc.; Waltham, MA, USA) at 5000 per well and incubated for 24 h in 5% CO_2_ humidified incubator at 37 °C. VSMC proliferations were determined using the WST assay as described above.

### 3.7. Monocyte-Associated VSMC Trans-Well Migration Assay

VSMCs were seeded at 5 × 10^4^ per well onto 8-µm Transwell inserts (Corning Inc.; Corning, NY, USA). Lower chambers were filled with 500 μL of containing each CM and incubated for 48 h in a 5% CO_2_ atmosphere. Migration activities were evaluated using the WST assay as described above.

### 3.8. Western Blotting

Inhibitory effects of DK, PHB, PFFA and PPB on monocyte-associated EC survival and VSMC proliferation and migration were investigated by western blotting. Cell lysates were prepared as described above. Equal amounts of proteins were separated by 8–12% sodium dodecyl sulfate polyacrylamide gel electrophoresis (SDS-PAGE) and then transferred to polyvinylidene fluoride (PVDF) membranes, which were incubated with appropriate diluted primary antibodies at 4 °C overnight. Membranes were then washed with tris buffered saline containing 1% Tween 20 (TTBS) three times and incubated with secondary antibodies for 1 h at room temperature. Primary and secondary antibodies are listed in Table S1. Membranes were developed by enhanced chemiluminescence (ECL) on LAS-4000s (GE Healthcare; Chicago, IL, USA).

#### AKT Inhibition Study

EC were seeded at a density of 10^5^ per well in 100 mm culture dish (SPL Life Science; Pocheon, Korea) and incubated for 24 h in 5% CO_2_ humidified incubator at 37 °C. Then ECs were treated with A6730 (Sigma) 40 µM for 1 h. 1 h later, the supernatant of monocyte was treated for 48 h. Then EC were isolated EzRIPA lysis kit (ATTO).

### 3.9. Quantitative Real Time Polymerase Chain Reaction (qRT-PCR)

qRT-PCR was performed using the CFX384 TouchTM Real-Time PCR detection system and reaction efficiencies and threshold cycle numbers were determined using CFX ManagerTM Software. Primers are detailed in Table S2.

### 3.10. Statistical Analysis

Non-parametric analysis was used given the small samples available. Comparisons were made using the Mann-Whitney U test. Significant differences are indicated as follows; by an asterisk (*) versus PBS, $ versus PA-BSA, and # versus PA-BSA with PPB. Results are presented as means ± SDs and experiments were performed in triplicate. The analysis was conducted using SPSS version 22 (IBM Co.; Armonk, NY, USA).

## 4. Conclusions

Four major phlorotannins, that is, DK, PHB, PFFA and PPB, were isolated for the ethanoic extraction of *E. cava*. Monocyte trans-migration and inflammatory macrophage differentiation by monocytes were effectively reduced by PPB, which also modulated vascular tone by protecting monocyte-associated EC death, by increasing phosphorylations of PI3K-AKT and AMPK and reducing monocyte-induced VSMC proliferation and migration via the phosphorylations of ERK and AKT in PPB treated the cells. The study suggests PPB be considered as a component in healthy functional foods to ameliorate vascular dysfunction in diseases associated with poor circulation.

## Supplementary Materials

The following are available online at http://www.mdpi.com/1660-3397/16/11/441/s1, Materials and methods: High-performance liquid chromatography (HPLC) chromatogram, Raw 264.7 cell cultivation and quantification of Western blotting; Figure S1: HPLC chromatograms and purity of four compounds from *E. cava* extract; Figure S2: Mass spectrometry analysis of four compounds from *E. cava* extract; Figure S3: Inhibitory effects of PPB in monocyte polarization and related cytokines; Figure S4: Inhibitory effects of PPB in Raw 264.7 cell-associated endothelial cell death; Figure S5: Inhibitory effects of PPB in Raw 264.7-associated VSMC proliferation and migration. Table S1: List of antibodies for western blotting; Table S2: List of primer for qRT-PCR.
