# Supplementary material for: Protective Effect of Pyrogallol-Phloroglucinol-6,6-Bieckol from Ecklonia cava on Monocyte-Associated Vascular Dysfunction
Source: Mar Drugs. 2018 Nov 9;16(11):441. doi: 10.3390/md16110441 (PMC6266154; doi:10.3390/md16110441)
Supplement: Supplementary file 1 [file marinedrugs-16-00441-s001.zip › Oh et al_Supple material for revision_2'Revision.docx]

**Supplementary Figure**

**
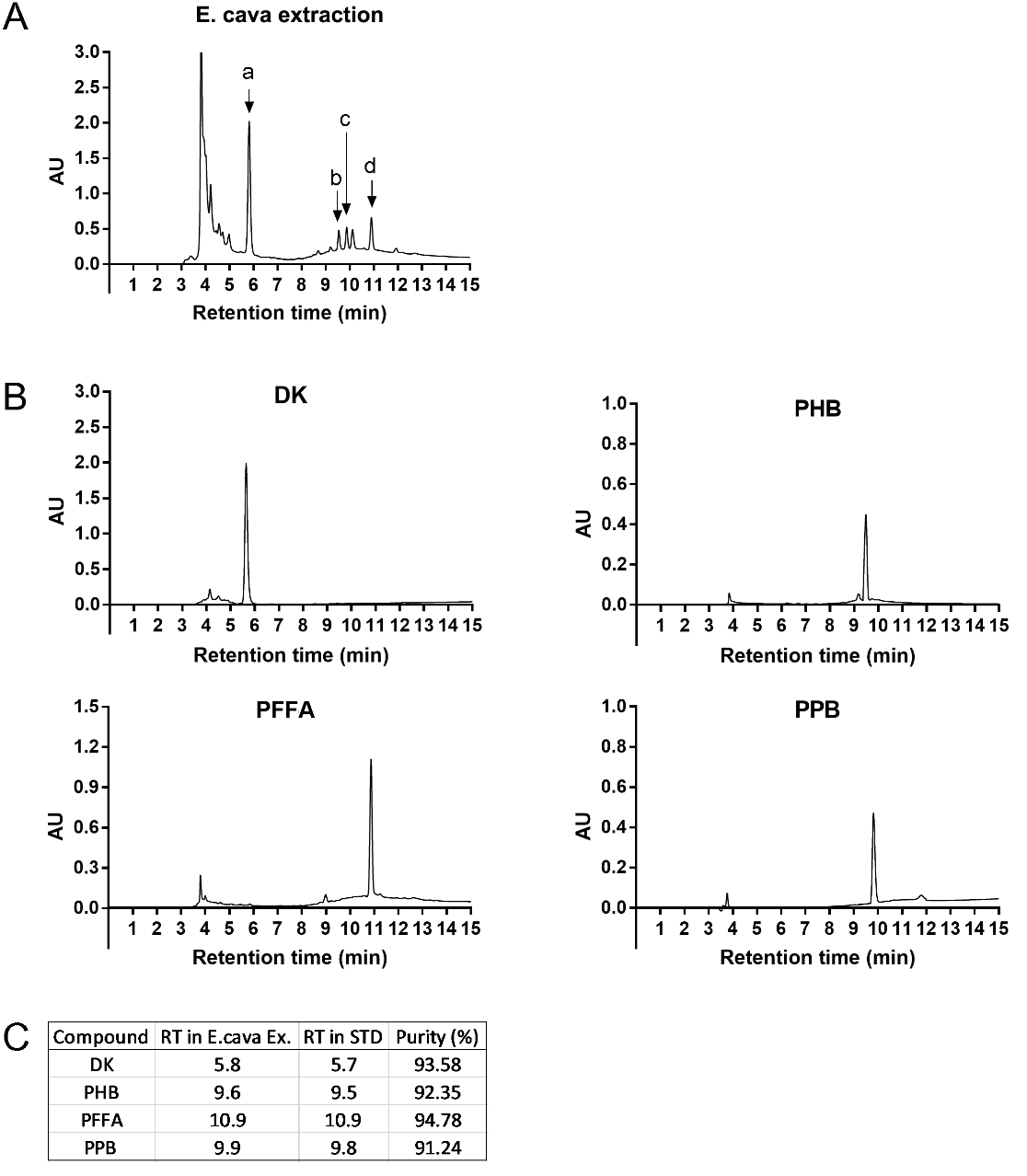
Supplementary Figure 1. HPLC chromatograms and purity of four phlorotannins from *E. cava* extract**

(A) HPLC chromatograms of *E. cava.* The peaks labeled a-d correspond to dieckol, PHB, PPB and PFFA, respectively and checked purity of them. a: dieckol, b: 2,7-phloroglucinol-6,6-bieckol (PHB), c: pyrogallol-phloroglucinol-6,6-bieckol (PPB), d: phlorofucofuroeckol-A (PFFA). (B) Separately isolated single phlorotannin was validated HPLC chromatograms. (C) Purity of isolated four phlorotannins from E. cava extract

**
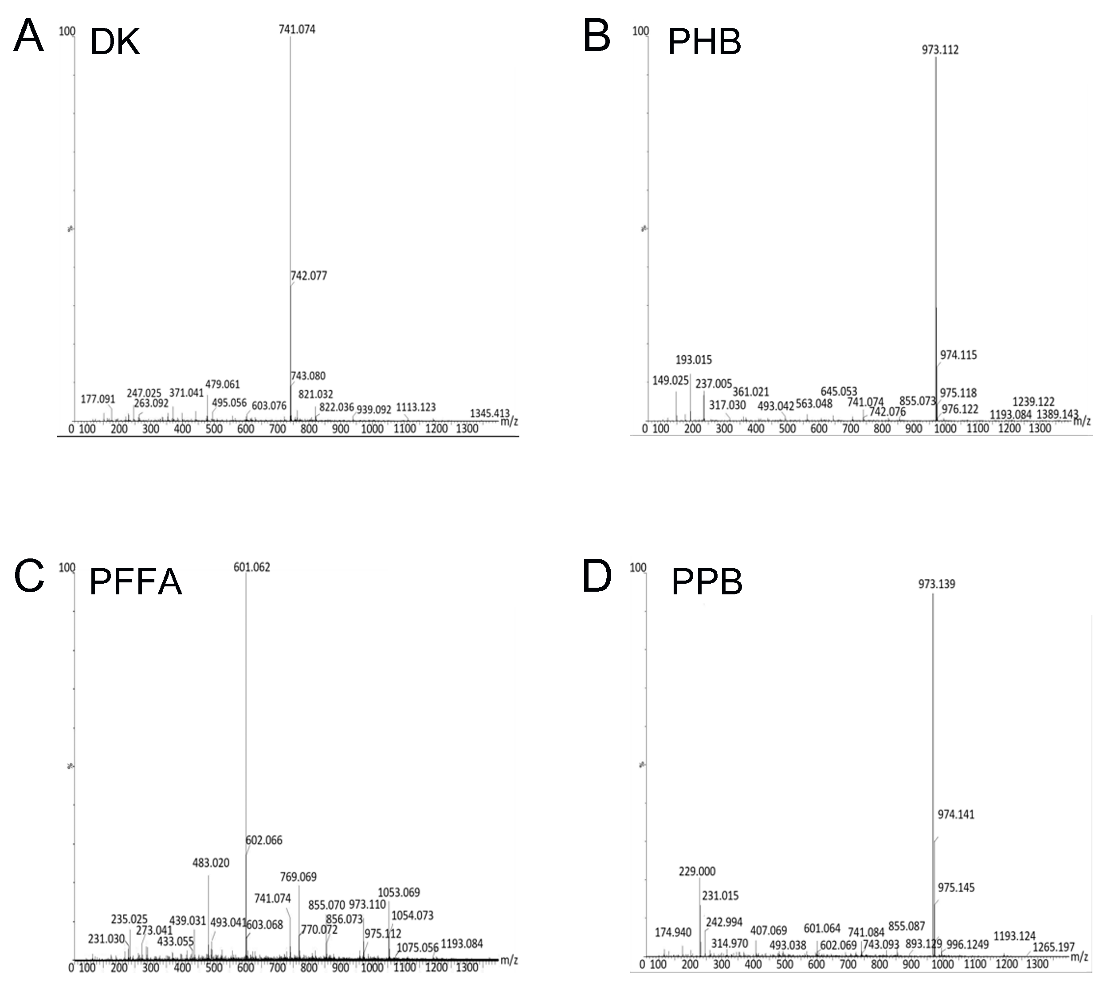
Supplementary Figure 2. Mass spectrometry analysis of four phlorotannins from *E. cava* extract**

ESI-MS spectra of isolated four phlorotannins. (A) DK (B) PHB (C) PFFA and (D) PPB were validated. DK: dieckol, PHB: 2,7-phloroglucinol-6,6-bieckol, PFFA: phlorofucofuroeckol-A, PPB: pyrogallol-phloroglucinol-6,6-bieckol

**
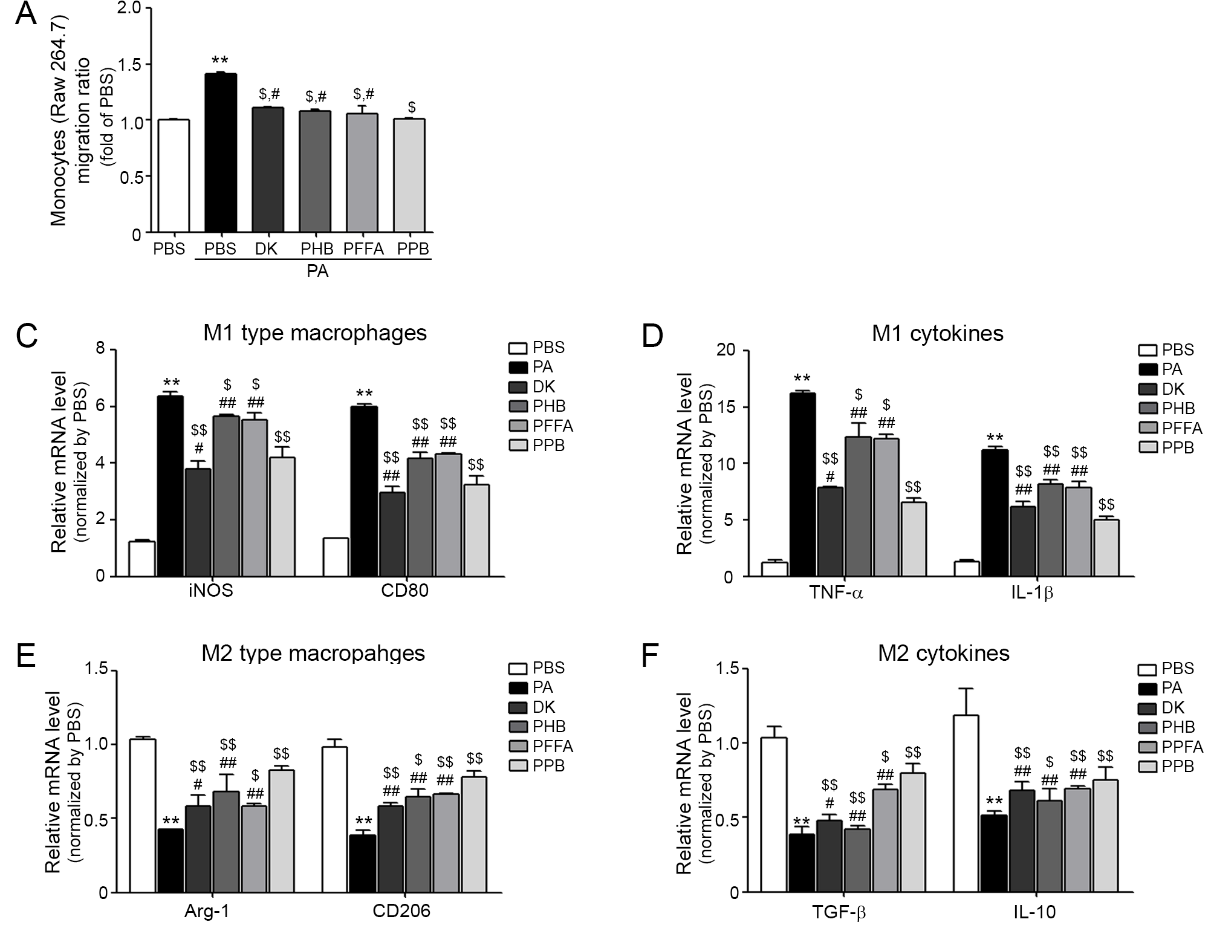
Supplementary Figure 3. Inhibitory effects of PPB in monocyte trans-migration, polarization and related cytokines**

(A) Migrating Raw 264.7 cell levels in 4 phlorotannins with PA-BSA as determined by the trans-well migration assay. (B, C) mRNA expression levels of M1 type macrophages marker (*iNOS* and *Cd80*) and M2 type macrophages marker (*Arg-1* and *Cd206*) as determined by qRT-PCR. (D, E) mRNA expression levels of M1 type macrophages related cytokines (*TNF-α* and *IL-1β*) and M2 type macrophages related cytokines (*TGF-β* and *IL-10*) by qRT-PCR. **, P < 0.01, ***, P < 0.001, vs. PBS; $, P < 0.05, $$, P < 0.01, vs. PA-BSA; #, P < 0.05, ##, P < 0.01, vs. PA-BSA with PPB, DK; dieckol, PHB; 2,7-phloroglucinol-6,6-bieckol, PFFA; phlorofucofuroeckol-A, PPB; pyrogallol-phloroglucinol-6,6-bieckol.

**
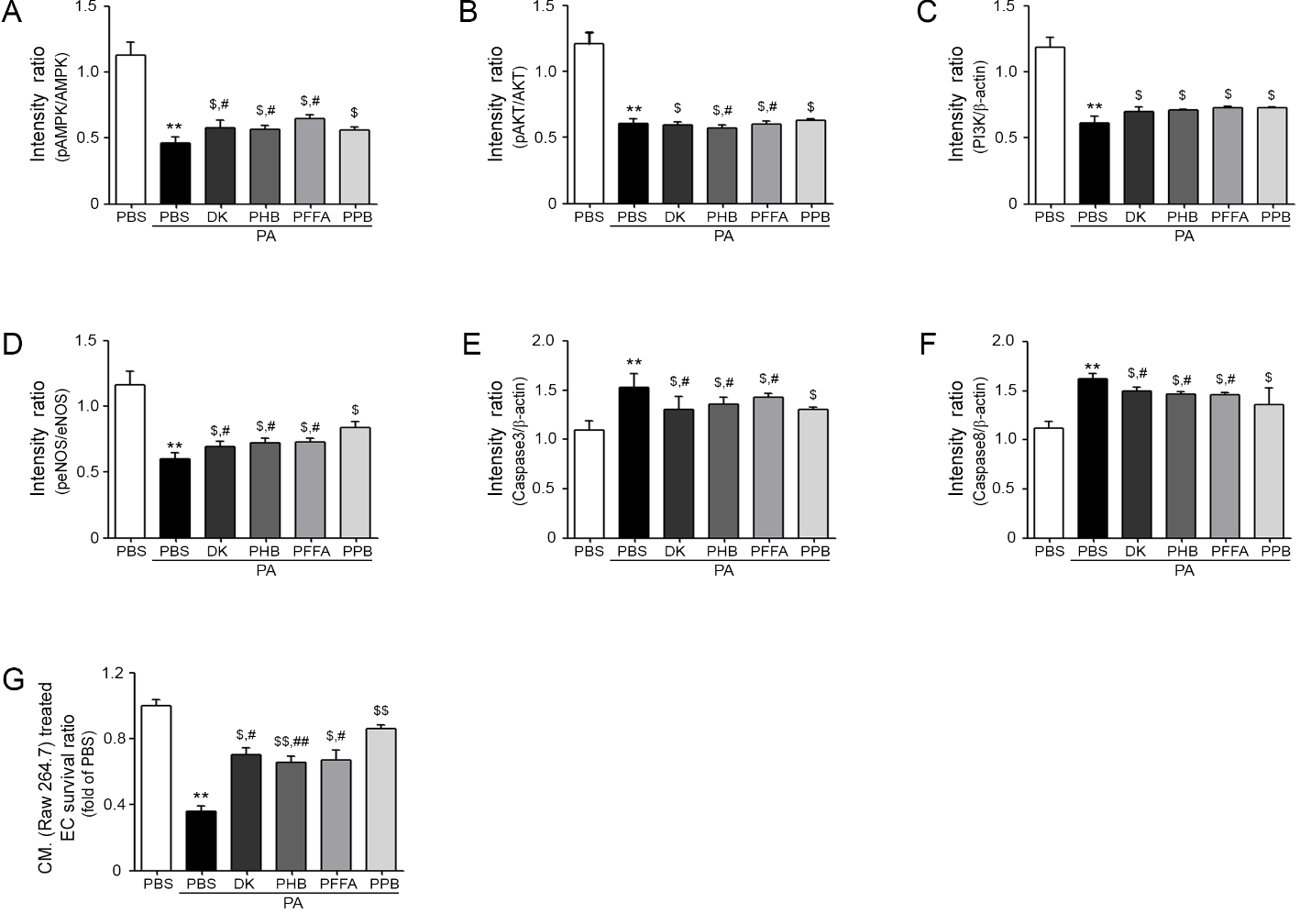
Supplementary Figure 4. Inhibitory effects of PPB in Raw 264.7 cell-associated endothelial cell death**

(A-F) Protein level graph shows quantified protein levels of cell-death related molecules, that is, (B) pAMPK/AMPK, (C) pAKT/AKT, (D) PI3K (E) peNOS/eNOS, (F) Caspase 3, and (G) Caspase 8. (G) Conditioned medium (CM) collected from PA-BSA with or without four phlorotannins treated Raw 264.7 cell. ECs were validated after CM treatment by a cell survival assay. *, P < 0.05, **, P < 0.01, ***, P < 0.001, vs. PBS; $, P < 0.05, $$, P < 0.01, $$$, P < 0.001, vs. PA-BSA; #,P < 0.05, ##, P < 0.01, ###, P < 0.001 vs. PA-BSA with PPB, DK; dieckol, PHB; 2,7-phloroglucinol-6,6-bieckol, PFFA; phlorofucofuroeckol-A, PPB; pyrogallol-phloroglucinol-6,6-bieckol.

**
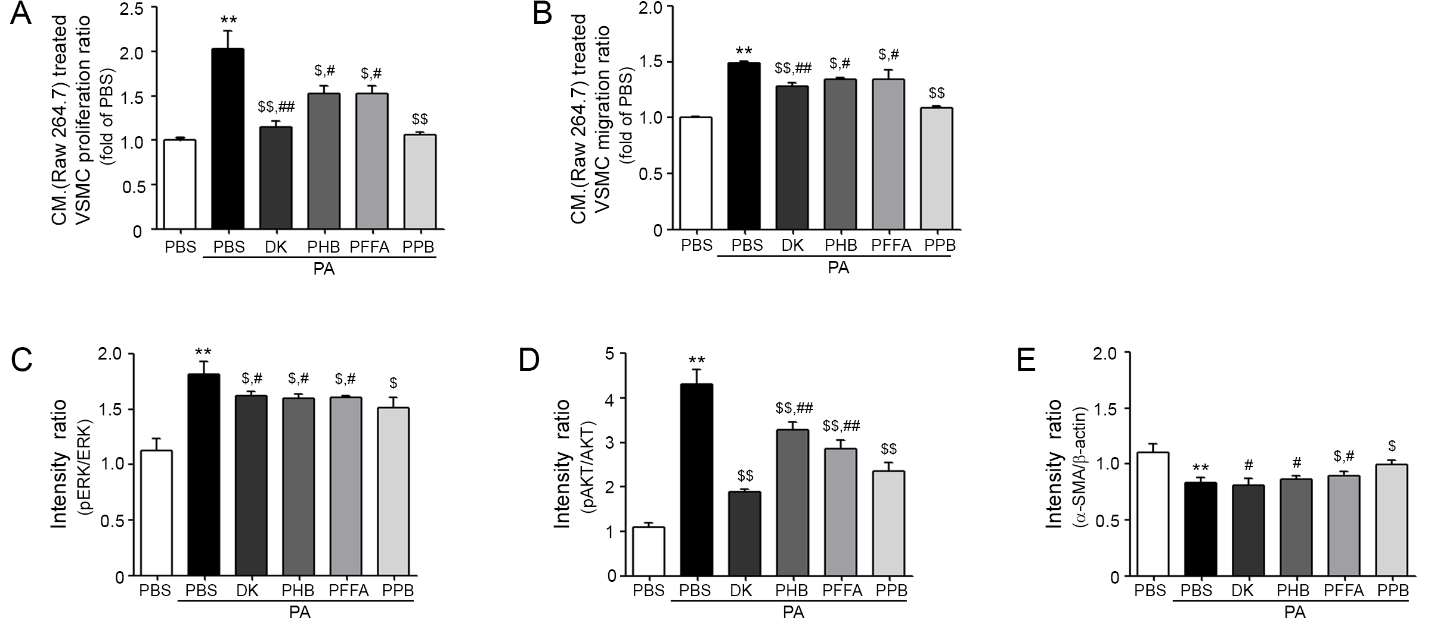
Supplementary Figure 5. Inhibitory effects of PPB in Raw 264.7-associated VSMC proliferation and migration**

(A) Conditioned medium (CM) collected from PA-BSA with or without four phlorotannins treated Raw 264.7 cell. VSMC proliferation were measured using a proliferation assay after CM treatment (B) Trans-migrating VSMC numbers were measured using a trans-migration assay. (C-E) Protein level graph shows quantified protein levels of proliferation and migration related molecules, that is, (C) pERK/ERK, (D) pAKT/AKT, (E) α-SMA. **, P < 0.01, ***, P < 0.001, vs. PBS; $, P < 0.05, $$, P < 0.01, vs. PA-BSA; #,P < 0.05, ##, P < 0.01, vs. PA-BSA with PPB, DK; dieckol, PHB; 2,7-phloroglucinol-6,6-bieckol, PFFA; phlorofucofuroeckol-A, PPB; pyrogallol-phloroglucinol-6,6-bieckol.

**Supplementary Tables**

**Supplementary Table 1. List of antibodies for Western blotting**

| **Antibody name** | **Company** | **Cat. No.** | **Dilution rate** |
| --- | --- | --- | --- |
| β-actin | Cell signaling | 4967s | 1:1,000 |
| ERK | Cell signaling | 9102s | 1:1,000 |
| Phospho-ERK | Cell signaling | 9101s | 1:1,000 |
| AKT | Abcam | 81283S | 1:1,000 |
| Phospho-AKT | Ab5076 | Ab5076 | 1:5,000 |
| AMPK | Abcam | Ab207442 | 1:1,000 |
| Phospho-AMPK | Abcam | Ab133448 | 1:1,000 |
| eNOS | Thermo Fisher | PA3-031A | 1:1,000 |
| Phospho-eNOS | Cell signaling | 9571 | 1:1,000 |
| PI3K | FineTest | FNab06422 | 1:1,000 |
| α-Smooth muscle actin | Abcam | Ab5694 | 1:1,000 |
| Caspase 3 | Cell signaling | 9662 | 1:1,000 |
| Caspase 8 | Cell signaling | 9746 | 1:1,000 |

**Supplementary table 2. List of primer for qRT-PCR**

| **Gene** | | **Primers** |  |
| --- | --- | --- | --- |
| *β-actin* | Forward | 5'-ACA AAG CTG TTC AGT GTC TCC A-3’ | |
|  | Reverse | 5'-CTC CGT TTC CAG AAT ACA CAC A-3’ | |
| *iNOS* | Forward | 5'- CACAGCAATATAGGCTCATCCA-3’ | |
|  | Reverse | 5'- AGCCTCATGGTAAACACGTTCT-3’ | |
| *CD80* | Forward | 5'-GACCGAATCTACTGGCAAAAAC-3’ | |
|  | Reverse | 5'-TTCTTATACTCGGGCCACACTT-3’ | |
| *CD206* | Forward | 5'-TGTATTCTTTGCCTTTCCCAGT-3’ | |
|  | Reverse | 5'-GATAAAAGCCAGAAGCAGGAGA-3’ | |
| *Arg-1* | Forward | 5'-ACAGAACTAAGCAAACGCCTTC-3’ | |
|  | Reverse | 5'-AGAAAGGAACTGCTGGGATACA-3’ | |
| *TNF-α* | Forward | 5'-TTCTGTCTACTGAACTTCGGGGTGATCGGTCC-3’ | |
|  | Reverse | 5'-GTATGAGATAGCAAATCGGCTGACGGTGTGGG-3’ | |
| *IL-1β* | Forward | 5'-CTTTTCGTGAATGAGCAGACAG-3’ | |
|  | Reverse | 5'-TCAGCTTCAATGAAAGACCTCA-3’ | |
| *TGF-β* | Forward | 5'- CTGGCAGTAGCTCCCCTATTTA-3’ | |
|  | Reverse | 5'- ACCAGGGTAAAAATCGAGATGA-3’ | |
| *IL-10* | Forward | 5'-ATGGTGTCCTTTCAATTGCTCT-3’ | |
|  | Reverse | 5'-AGGATCTCCCTGGTTTCTCTTC-3’ | |
| *E-selectin* | Forward | 5'-ATGAAATGTCTTCCCAGTGCTT-3’ | |
|  | Reverse | 5'-TGATCCCTTCAGTTCAAATCCT-3’ | |
| *ICAM-1* | Forward | 5'-ATAACCGCCAGAGAAAGATCA -3’ | |
|  | Reverse | 5'-GGCTTGTCCCTTGAGTTTTATG-3’ | |
| *VCAM-1* | Forward | 5'- GAGACCTGTCACTGTCAACTGC -3’ | |
|  | Reverse | 5'- CATCAGTGTAGTCTCCCCCTTC -3’ | |
| *vWF* | Forward | 5'-AAAGCTCCAGCAAGTTGAAGAC-3’ | |
|  | Reverse | 5'-CATCCACACAAACTCCAGAAAA-3’ | |

**Supplementary materials and methods**

**High-performance liquid chromatography**

A reverse phase High-performance liquid chromatography (HPLC) system comprising an Poroshell 120 C18 column (4 μm, 4.6 × 150 mm; Agilent) was used to further purify the selected fraction (Alliance 2695; Waters Corp., MA, USA). This was connected to a photodiode array detector (Waters Corp). Gradient elution was performed with 0.1% Formic acid (FA) and water as the eluents. Between 0 min and 2 mins, 43% of the FA and methanol and to 8 mins, 70% of the FA and methanol and to 15 mins 100% of the FA and methanol and to 30 mins 43% of the FA and methanol were used. The column flow rate was maintained at 0.4 mL/min and injection volume was 10 µL. We finally confirmed purity of four phlorotannins is more than 90% was used in the study.

**Mass spectrometry analysis**

This analysis was performed following previous study [1]. Briefly, Electrospray ionization (ESI)/ spectrometry (MS) analyses were performed using a Hewlett-Packard 1100 series HPLC system equipped with a DAD detector, a degasser, an auto-sampler, a column oven and a binary pump (Hewlett-Packard; Waldbronn, Germany) coupled to a Finnigan MAT LCQ ion-trap mass spectrometer (Finnigan MAT; CA, USA). The MS was equipped with a Finnigan electrospray source and was capable of analyzing ions up to 2000 m/z. Negative ion mass spectra of the column elute was recorded in the range 100-2000 m/z. The source voltage was set to 5 kV and the capillary temperature to 275 ℃. The other conditions were as follows: sheath gas, 80 psi (551.6 kPa); inter-octapole lens voltage, 10 V; capillary voltage, -36.5 V; auxiliary gas, 20 psi (137.9 kPa).

**Raw 264.7 cells cultivation**

Raw 264.7 cells for monocyte/macrophage cell line were purchased from Korea cell bank (Republic of Korea). Dulbecco's Modified Eagle's medium (DMEM; Hyclone) and 1% penicillin-streptomycin (Gibco) were used as growth medium. To investigate the inhibitory effects of DK, PHB, PFFA and PPB in 0.25 mM PA-BSA treated Raw 264.7 cells, we used the same concentration (2.5 ug/ml) for a treatment time of 48 hrs. To collect conditioned medium (CM), Raw 264.7 cells were treated with PA-BSA with or without DK, PHB, PFFA or PPB for 48 hrs.

**Quantification of Western blotting results**

The Western blotting band was quantified by image J program. PI3K, Caspase 3, Caspase 8 and α-SMA were normalized b-actin. phospho-form proteins including pAMPK-AMPK, pAKT-AKT, peNOS-eNOS were normalized total-form protein.

**Supplementary reference**

1. Lee, J.H.; Ko, J.Y.; Oh, J.Y.; Kim, C.Y.; Lee, H.J.; Kim, J.; Jeon, Y.J. Preparative isolation and purification of phlorotannins from Ecklonia cava using centrifugal partition chromatography by one-step. *Food Chem.* **2014**, 158, 433-437.
