# Supplementary figures and images for: Protective Effect of Pyrogallol-Phloroglucinol-6,6-Bieckol from Ecklonia cava on Monocyte-Associated Vascular Dysfunction
Source: Mar Drugs. 2018 Nov 9;16(11):441. doi: 10.3390/md16110441 (PMC6266154; doi:10.3390/md16110441)

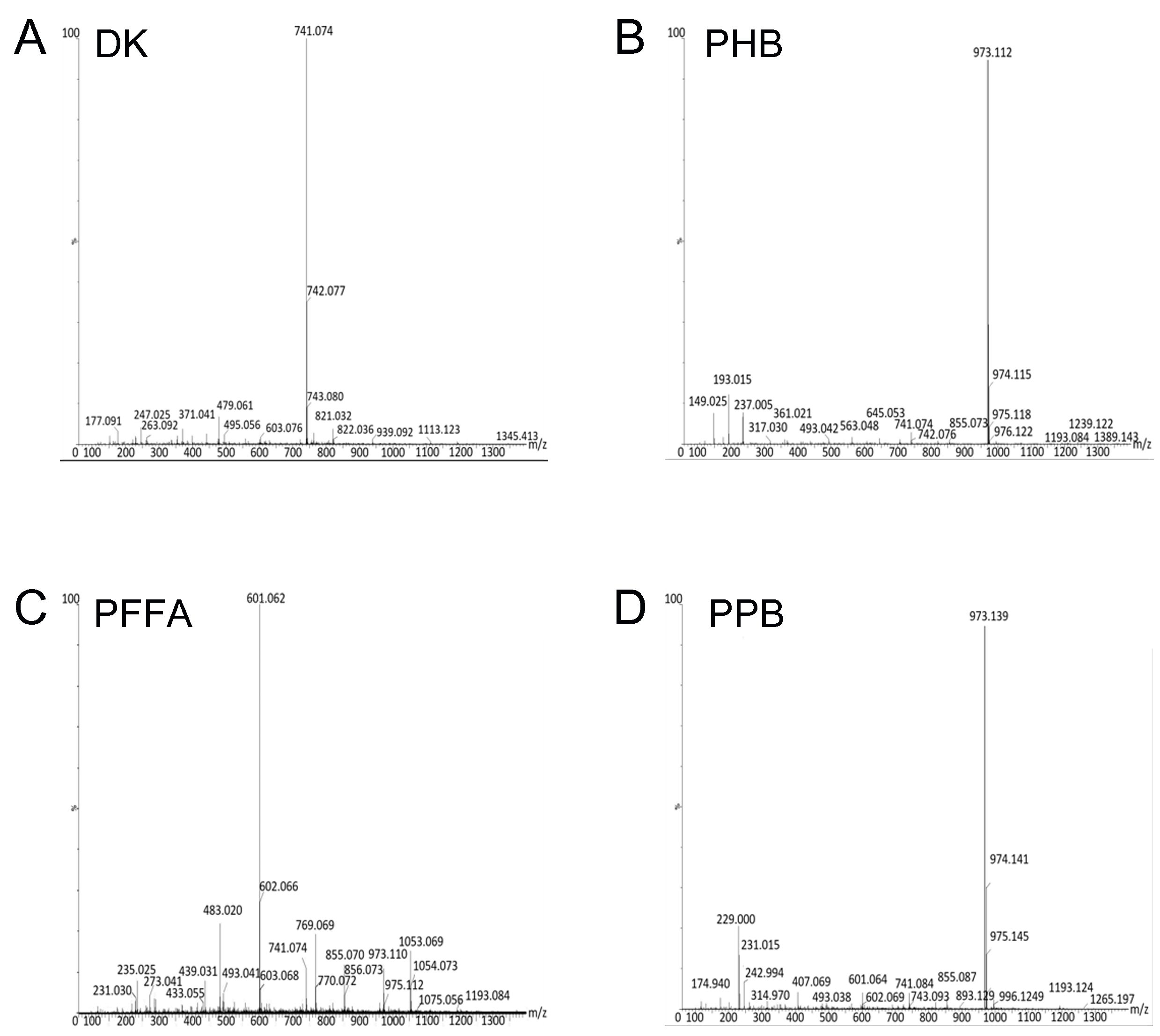

Supplement: Supplementary file 1 [file marinedrugs-16-00441-s001.zip › MD_sF2_SY.tif]

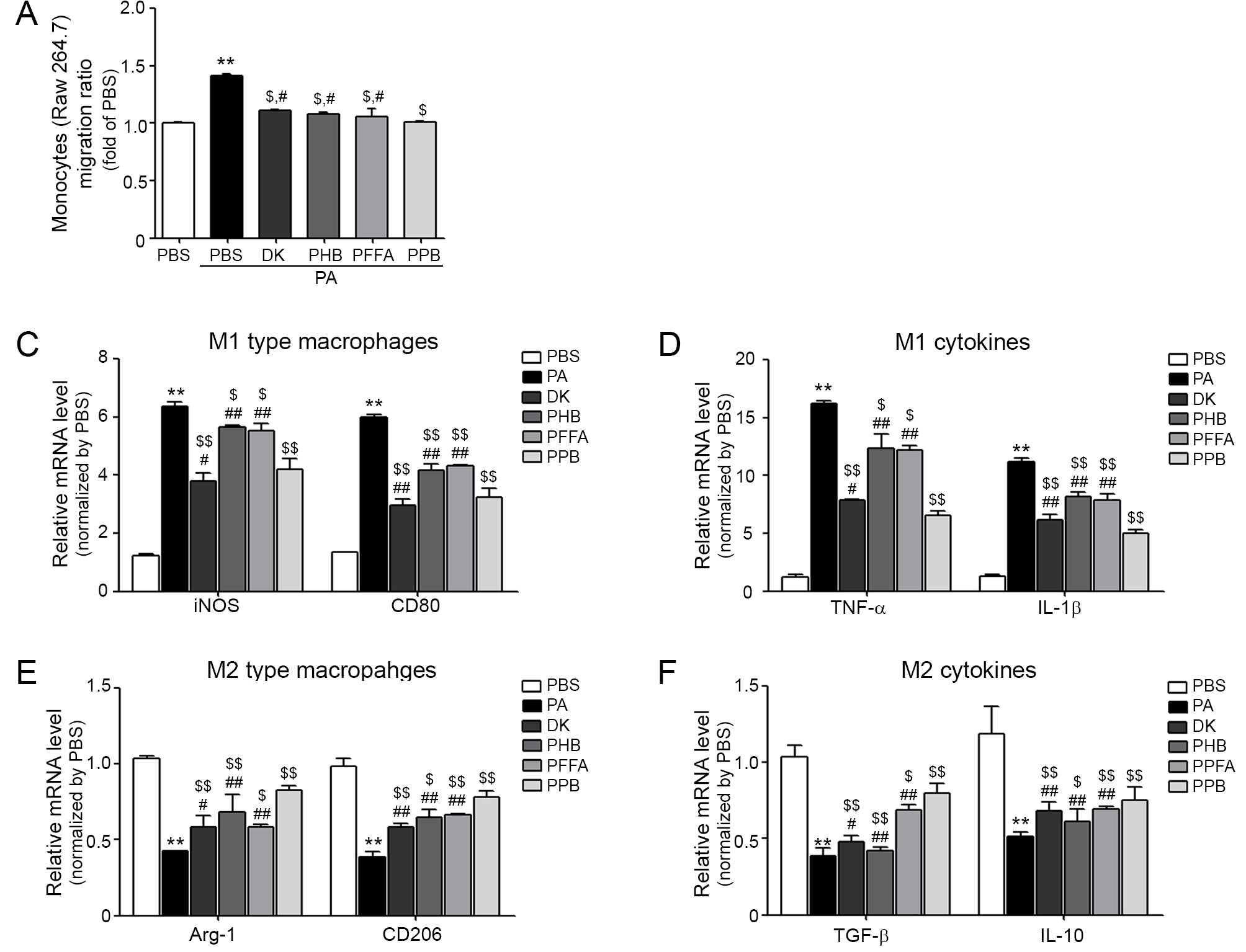

Supplement: Supplementary file 1 [file marinedrugs-16-00441-s001.zip › MD_sF3_SY.tif]

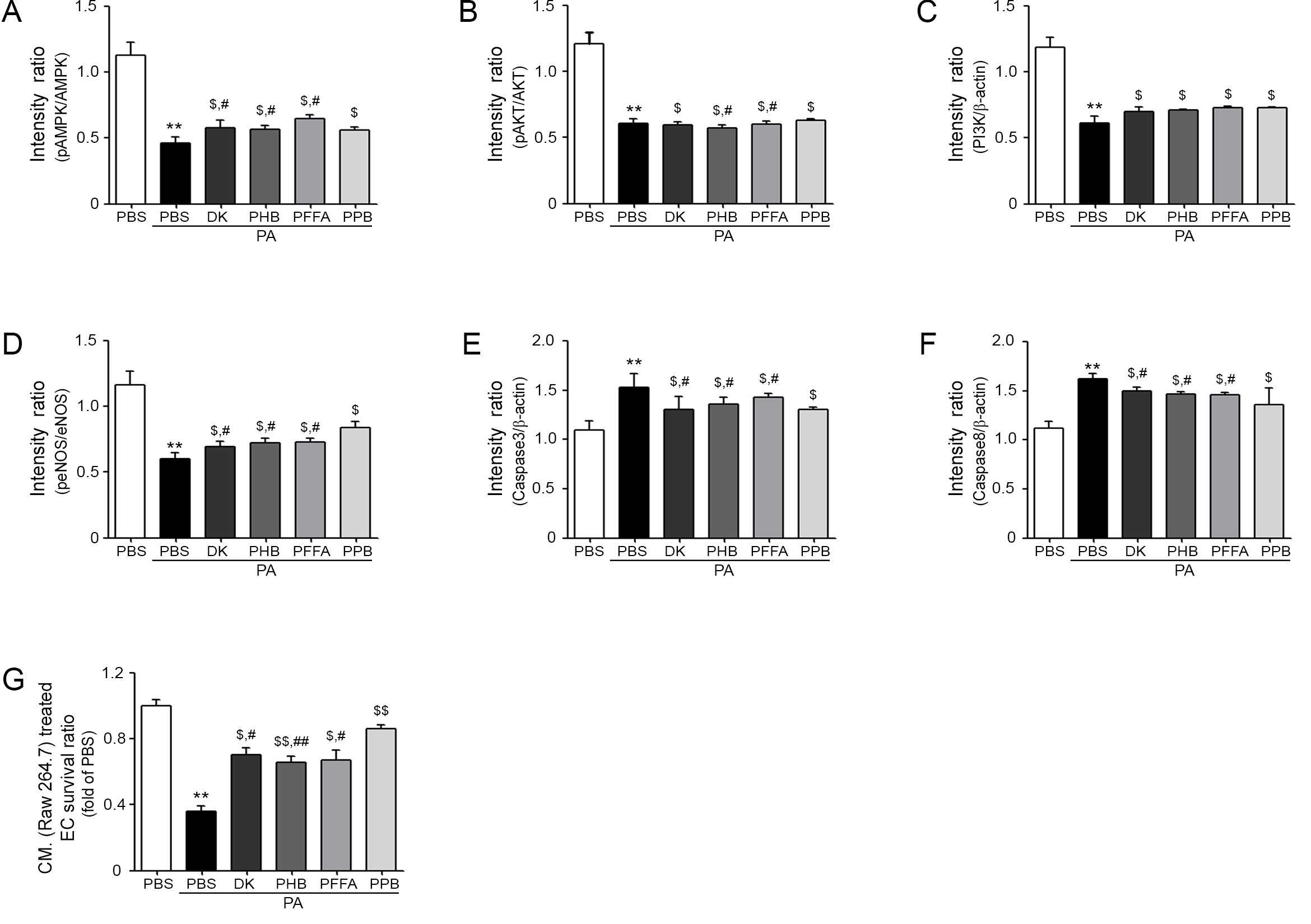

Supplement: Supplementary file 1 [file marinedrugs-16-00441-s001.zip › MD_sF4_SY.tif]

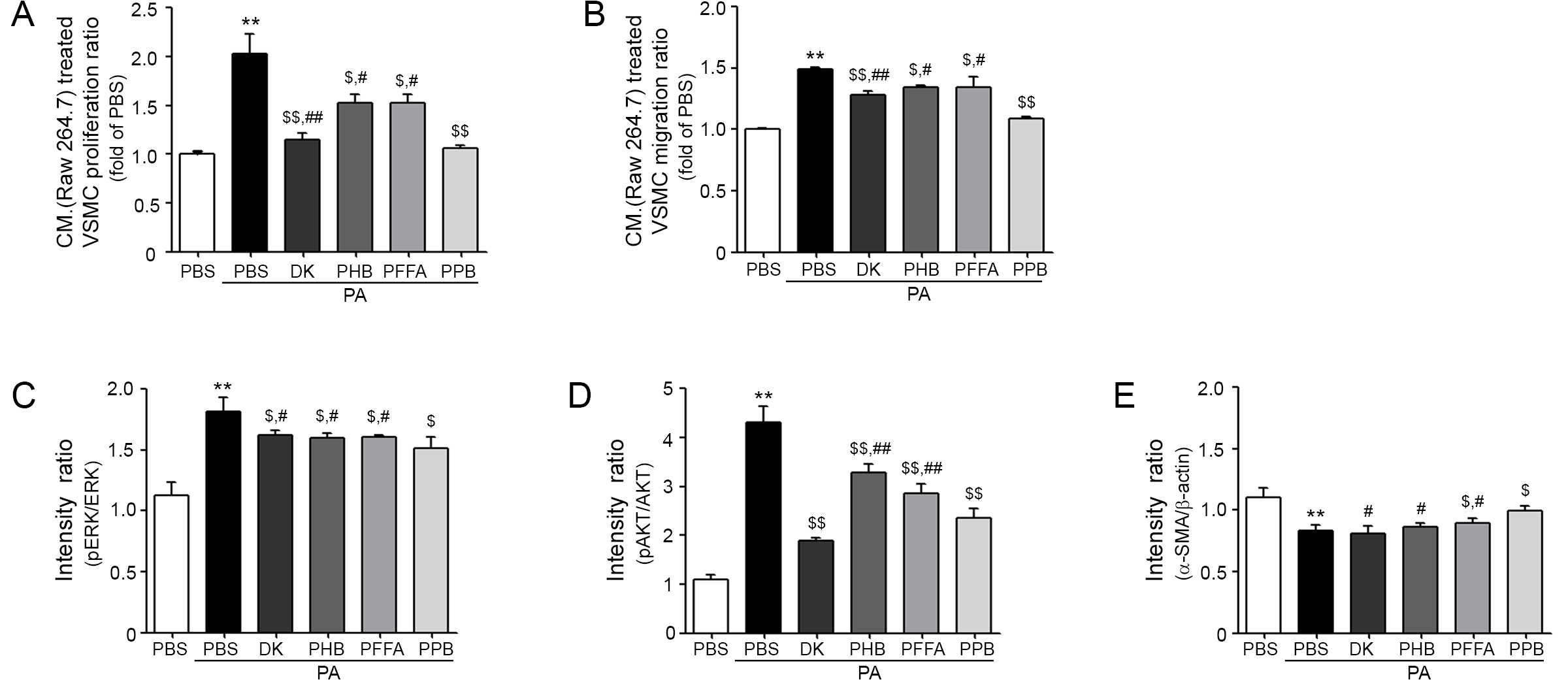

Supplement: Supplementary file 1 [file marinedrugs-16-00441-s001.zip › MD_sF5_SY.tif]

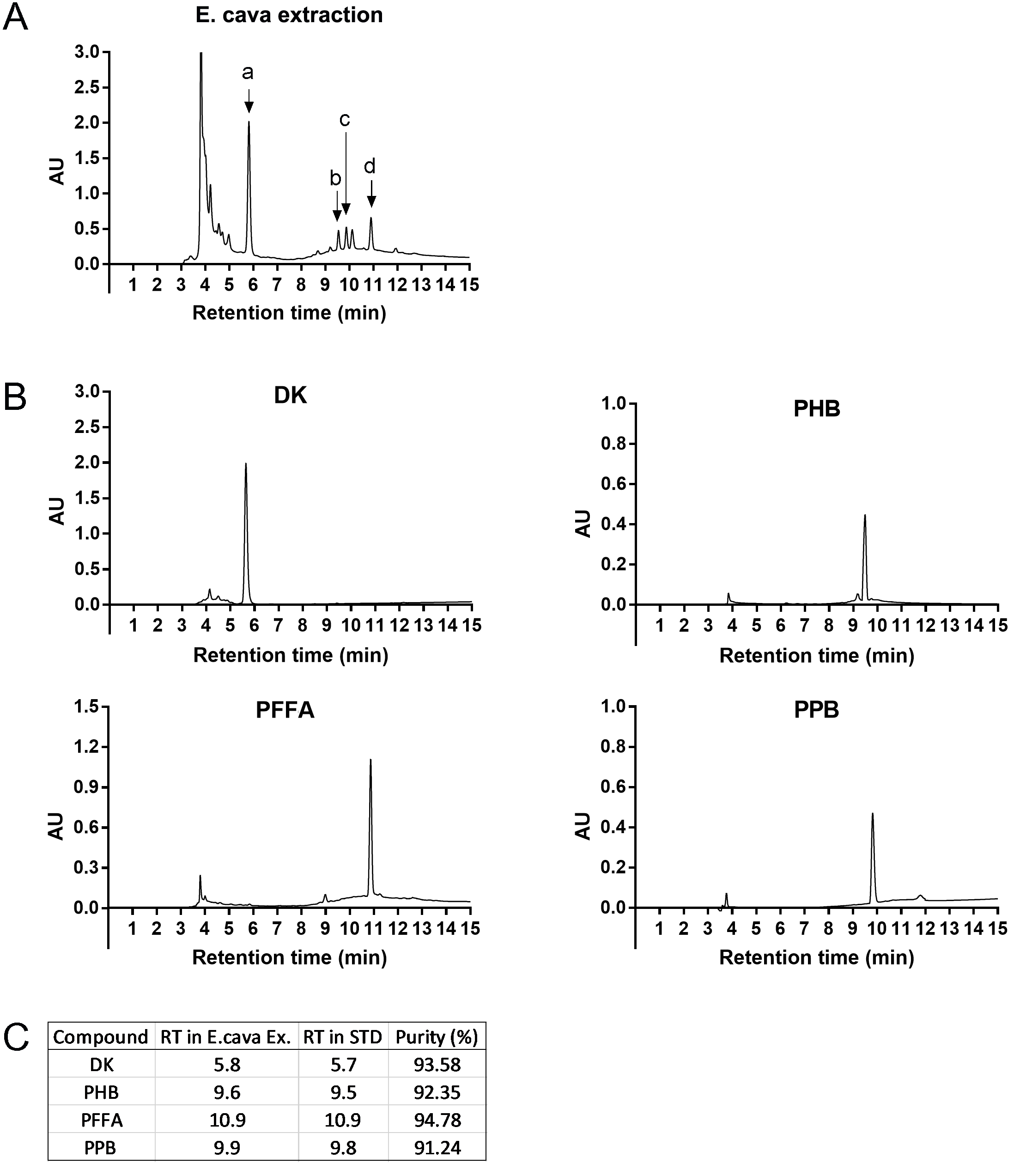

Supplement: Supplementary file 1 [file marinedrugs-16-00441-s001.zip › MD_sF1_SY.tif]
